# Network Biology and Translational Strategies in Liver Fibrosis

**DOI:** 10.3390/biomedicines14030730

**Published:** 2026-03-23

**Authors:** Takefumi Kimura, Takanobu Iwadare, Shun-ichi Wakabayashi

**Affiliations:** 1 Department of Medicine, Division of Gastroenterology, Shinshu University School of Medicine, 3-1-1 Asahi, Matsumoto 390-8621, Japan; 2 Consultation Center for Liver Diseases, Shinshu University Hospital, Matsumoto 390-8621, Japan; 3 Institute for Biomedical Sciences, Research Cluster for Social Implementation, Matsumoto 390-8621, Japan

This Editorial introduces the Special Issue titled *“Liver Fibrosis: Molecular Mechanisms, Potential Therapeutic Targets and Clinical Biomarkers”* in *Biomedicines*. Liver fibrosis is a dynamic and potentially reversible wound-healing response to chronic hepatic injury [1]. However, despite decades of mechanistic progress, no dedicated anti-fibrotic therapy has yet achieved regulatory approval. The studies collected in this Special Issue collectively demonstrate that fibrosis research is increasingly moving beyond descriptive histology toward mechanistic immune–metabolic stratification and translational precision medicine.

## 1. Immune Reprogramming Defines Advanced Fibrosis

A major advance highlighted in this issue is the identification of a fibrosis-stage-specific immune signature [2]. In a cohort of 77 human liver tissues spanning fibrosis stages F1 to F4, immune-focused gene profiling identified a five-gene signature—CHIT1, FCER1G, OSM, VEGFA, and ZAP70—that distinguished advanced fibrosis (F3–F4) from mild fibrosis (F1–F2) with an accuracy of 94.8%, sensitivity of 96.2%, and specificity of 91.7%. Notably, CHIT1 expression increased in advanced fibrosis, consistent with macrophage-dominant fibrogenic remodeling, whereas FCER1G, OSM, VEGFA, and ZAP70 showed relative downregulation, reflecting a shift away from immune activation pathways that are prominent during earlier inflammatory stages. This suggests that advanced fibrosis represents not simply “more inflammation” but rather a qualitatively distinct immune-reprogrammed state. Importantly, network analysis revealed STAT3 as a highly connected signaling hub interacting with the signature genes, identifying a potentially druggable pathway in advanced disease.

Complementing this molecular analysis, a detailed review of natural killer (NK) cells in fibrosis underscores their dual function [3]. NK cells can eliminate activated HSCs via NKG2D-dependent cytotoxicity and TRAIL-mediated apoptosis. However, NK activity is suppressed in advanced fibrosis through regulatory T-cell interactions and altered cytokine environments. The review highlights therapeutic strategies—including enhancement of activating receptor signaling and modulation of NK metabolism—that could restore anti-fibrotic immunity. Together, these studies are consistent with the emerging view that immune recalibration, rather than blanket immunosuppression, may be required for effective anti-fibrotic strategies [4,5].

## 2. Neurohumoral Signaling: Targeting the RAAS–NP Axis

Beyond immune regulation, systemic neurohumoral pathways influence fibrogenesis. In a murine CCl_4_-induced fibrosis model, combined angiotensin receptor blockade and neprilysin inhibition significantly attenuated fibrosis [6]. Treatment reduced hepatic hydroxyproline content, decreased α-SMA-positive HSC expansion, and suppressed pro-fibrogenic gene expression. Mechanistically, sacubitril increased circulating atrial and C-type natriuretic peptides, activating guanylate cyclase-A/cGMP/PKG signaling and suppressing TGF-β-induced fibrogenic responses in LX-2 cells. Concurrently, valsartan inhibited angiotensin II-stimulated proliferation via AT1 receptor blockade. These findings illustrate how rebalancing the profibrotic RAAS pathway and anti-fibrotic natriuretic peptide signaling can modulate stellate cell activation. Importantly, these agents are already clinically approved in cardiovascular disease, supporting the feasibility of therapeutic repurposing.

## 3. Metabolic Reprogramming and Precision Response Prediction

Metabolic dysfunction is a major driver of fibrosis in steatotic liver disease [7,8]. A preclinical study demonstrated that a clinically relevant dose (0.1 mg/kg/day) of a selective peroxisome proliferator-activated receptor α (PPARα) modulator reduced serum triglyceride levels while selectively activating hepatic PPARα without evidence of hepatotoxicity [9]. This dosing achieved plasma exposure comparable to human therapeutic levels and was associated with enhanced fatty acid β-oxidation and increased fibroblast growth factor 21 (FGF21) expression, supporting a targeted metabolic mechanism.

Translating these findings into clinical practice, a retrospective analysis of 88 patients with hypertriglyceridemic fatty liver disease treated for six months identified predictors of treatment response [10]. Responders were defined by a ≥30% reduction in alanine aminotransferase (ALT). Female sex, AST ≥ 45 U/L, ALT ≥ 60 U/L, and fat mass ≥37% were significantly associated with biochemical response, suggesting that baseline metabolic and inflammatory profiles influence treatment sensitivity. Notably, treatment efficacy in this study was assessed using biochemical improvement rather than direct measures of fibrosis regression. Similar biochemical improvements have also been reported in related studies, supporting the reproducibility of these metabolic effects [11,12,13]. Whether such responses translate into meaningful anti-fibrotic outcomes, however, remains to be established, underscoring the need for future studies with fibrosis-specific endpoints.

## 4. Translational Models That Mirror Human Disease

A persistent limitation in fibrosis research has been the poor translational fidelity of rodent models. A rabbit model of diet-induced non-alcoholic steatohepatitis described in this issue demonstrated steatohepatitis by 8 weeks and advanced fibrosis by 14 weeks, along with hypercholesterolemia and aortic atherosclerosis [14]. Unlike rodents, rabbits exhibit lipoprotein metabolism more similar to that in humans. This integrated NASH–atherosclerosis model better reflects the cardiometabolic context of human metabolic liver disease and may enhance the translational relevance of preclinical drug evaluation.

## 5. Oxidative Stress and Angiogenesis: Pleiotropic Modulation in Preclinical Models

Oxidative stress and angiogenesis are interwoven drivers of fibrogenesis [15]. In a toxin-induced fibrosis model, the administration of apigenin—a plant-derived flavonoid with pleiotropic biological activity—significantly reduced serum AST, ALT, and bilirubin levels; restored antioxidant defenses (increased glutathione and catalase and decreased malondialdehyde); and suppressed inflammatory cytokines including IL-1β, IL-6, and TNF-α [16]. In parallel, VEGF and CD34 expression were reduced, indicating the attenuation of pathological angiogenesis. Together, these findings illustrate how the simultaneous modulation of oxidative stress, inflammatory signaling, and angiogenic pathways by a single agent can attenuate fibrogenic processes in animal models, while emphasizing the need for validation in human studies to determine whether such pleiotropic effects translate into clinically meaningful anti-fibrotic efficacy.

## 6. Hepatic–Renal Interconnection in Metabolic Disease

Epidemiological and biological evidence supports a close relationship between metabolic-associated fatty liver disease (MAFLD) and diabetic kidney disease [17]. Meta-analyses indicate that MAFLD is associated with an approximately 40% increased risk of incident chronic kidney disease. In patients with type 2 diabetes, MAFLD has been linked to the development of DKD, with hazard ratios around 1.7. Importantly, the risk appears to increase with advancing liver fibrosis, as reflected by higher non-invasive fibrosis indices such as FIB-4.

However, causality has not been established. The review appropriately notes that shared metabolic risk factors—including insulin resistance, obesity, and systemic inflammation—may underlie the observed associations. Mechanistic hypotheses suggest that insulin resistance promotes lipotoxic stress, systemic inflammation contributes to endothelial dysfunction, and activation of the renin–angiotensin system may facilitate fibrogenic signaling in both hepatic and renal tissue. Whether these processes represent direct organ cross-talk or parallel consequences of systemic metabolic dysregulation remains unresolved.

## 7. Future Directions

Liver fibrosis research is entering a period of conceptual refinement. Taken together, the mechanistic, translational, and epidemiological studies presented in this Special Issue suggest three important transitions.

First, fibrosis may be better understood as immune reprogramming rather than persistent inflammation. Advanced fibrosis appears to represent a biologically distinct immune state, which may limit the effectiveness of uniform anti-inflammatory strategies. Second, anti-fibrotic therapy is increasingly recognized as requiring approaches beyond single-pathway inhibition. The convergence of neurohumoral, metabolic, vascular, and immune signaling on stellate cell activation highlights the challenges of reductionist strategies and supports the consideration of network-informed, combination approaches guided by molecular phenotyping. Third, fibrosis progression may be more appropriately viewed within a systemic metabolic–vascular context rather than as a purely organ-confined process. Evidence of inter-organ network dysfunction is consistent with the potential value of integrated, multi-system risk assessment.

Translating these insights into clinical impact will require continued alignment between disease biology, experimental modeling, biomarker development, and trial design. Whether a network-based framework can ultimately enable durable and clinically meaningful regression remains an open and important question for the coming decade.
